# Association of maternal folate intake during pregnancy with infant asthma risk

**DOI:** 10.1038/s41598-019-44794-z

**Published:** 2019-06-06

**Authors:** Weijian Li, Bo Xu, Yuepeng Cao, Yang Shao, Wanke Wu, Jun Zhou, Xiaofang Tan, Xiaoli Wu, Jing Kong, Chen Hu, Kaipeng Xie, Jiangping Wu

**Affiliations:** 1 The Affiliated Obstetrics and Gynecology Hospital of Nanjing Medical University, Nanjing Maternity and Child Health Care Hospital, Women’s Hospital of Nanjing Medical University, Nanjing, 210004 China; 2 Department of Urology, Drum Tower Hospital of Nanjing University Medical School, Nanjing, 210008 China; 3 State Key Laboratory of Reproductive Medicine, Institute of Toxicology, Nanjing Medical University, Nanjing, 211166 China; 4 The First People’s Hospital of Zhangjiagang city, The Zhangjiagang Affiliated Hospital of Soochow University, Suzhou, 215600 China

**Keywords:** Nutrition, Risk factors

## Abstract

Several studies assessed the association of maternal folate intake with infant asthma risk, but the findings are controversial. We performed a meta-analysis to clarify the association between maternal folate intake and infant asthma risk. PubMed and SCOPUS databases were searched for related studies published until August 2018. Fixed-effects models were applied to pool relative risks (RRs) and their corresponding 95% confidence intervals (CIs) due to the low heterogeneity. We also adopted generalized least-squares trend (GLST) estimation for the dose-response analysis. In our study, a total of 10 studies with maternal folate intake and 5 studies with blood folate concentration were included. We found that maternal folate intake during pregnancy was significantly related to the risk of infant asthma (RR = 1.11; 95% CI = 1.06–1.17). Similar results were found for geographic region from Europe (RR = 1.08; 95% CI = 1.01–1.16) and North America (RR = 1.20; 95% CI = 1.11–1.30) in subgroup analyses. Meanwhile, the dose-response analysis showed a linear relationship between maternal folic acid intake during pregnancy and infant asthma risk. This meta-analysis indicates that maternal folate intake during pregnancy could increase infant asthma risk. Therefore, the adverse effect of folic acid on infant asthma should not be ignored when it is supplemented during pregnancy to prevent birth defects.

## Introduction

Asthma is one of the common chronic respiratory diseases, with an estimated prevalence of 334 million worldwide^1^. Recently, the prevalence of asthma increased markedly among countries with Western lifestyles^2^. Asthma is also one of the top-10 chronic diseases for disability-adjusted life for 5- to 14-year-old children. Globally, asthma causes approximately 250,000 deaths a year, imposing a heavy burden on health system^3^. Therefore, the identification of risk factors for infant asthma is of major significance for early intervention and treatment of asthma^4,5^.

Asthma is considered to be the result of a combination of genetic and environmental risk factors. Changes in nutrition are linked to the development of asthma^6^. Folate, an essential B vitamin of nutrition, participates in the carrying and chemical activation (as tetrahydrofolates) of one-carbon units for further biosynthesis^7^. Such folate-mediated one-carbon metabolism plays a crucial role in purine and thymidylate synthesis, amino acid metabolism and S-adenosyl methionine formation^8^. Through this pathway, folic acid is involved in the synthesis of nucleic acids, methylation of DNA and regulation of cell growth^9^. Consequently, it plays an irreplaceable role in all of our activities in daily life, especially in the early stages of uterine growth and development^10^. Due to the growth of the foetus in utero, folate levels in pregnant women may be insufficient, which could lead to a number of birth defects, such as neural tube defect, growth retardation, cardiac defects and oral clefts^11–14^. Thus, the supplementation and fortification of food with folic acid are recommended among pregnant women^15^. In recent years, some countries have implemented mandatory folic acid fortifications^16^. However, high folate intake during pregnancy is considered to be the cause of some adverse effects in newborn and child health, such as large-for-gestational-age birth and respiratory illness^17,18^.

Given that pregnant women may consume a relatively higher dose of folic acid, the potential adverse effects on foetal development warrant evaluation. Emerging studies explored the relationship between maternal folic acid intake and the risk of childhood asthma. However, their results are not consistent. Therefore, we reviewed the available studies and performed a meta-analysis to better estimate the association between folate intake and childhood asthma risk.

## Methods

We followed the Meta-analysis of the Observational Studies in Epidemiology (MOOSE) guidelines when conducting and reporting this meta-analysis^19^.

### Search strategy

We searched PubMed and SCOPUS databases for relevant studies published through 1 August 2018. The SCOPUS database is an abstract and citation database containing all of the EMBASE databases^20^. We used the following keywords: “folate” OR “folic acid” in combination with “maternal” OR “pregnancy” in combination with “asthma”. No language or time restrictions were applied. A manual was used for all references of qualified research to identify other potentially relevant studies.

### Selection criteria

We included the eligible studies according to the following criteria: (1) the study was peer-reviewed original research; (2) the study was a cohort study; (3) the study provided the risk estimates of asthma associated with maternal folate intake or maternal folate concentration during pregnancy. Studies that provided only a crude estimate were excluded. For articles including the same study, the latest one was selected.

### Data extraction and quality assessment

For the included studies, study information, participants, exposure and outcome measurements, effect sizes and related statistics were extracted by two investigators. Disagreements were resolved through discussions. The quality of the eligible studies was assessed by the Newcastle-Ottawa Scale (NOS)^21^. For each cohort study, the highest score was 9 stars, and studies with 6 or more stars were considered to be of high quality.

### Statistical analysis

Multivariable-adjusted odds ratios (ORs), prevalence rates (PRs) or relative risks (RRs) with 95% confidence intervals (95% CIs) were included in the meta-analysis. For studies that provided multiple exposure periods of maternal folate intake, we chose the first trimester because the first trimester is the most critical period of DNA methylation during pregnancy^22^. For studies that provided RRs with 95% CIs from different lengths of follow-up for asthma, we chose the RRs from the longest length of follow-up for the outcome. Statistical heterogeneity across studies was estimated via the χ^2^-based Q-statistic, and we considered *P* < 0.05 to indicate significant heterogeneity. We conducted stratified analyses to search for potential differences in RRs in subgroups by exposure assessment, folate source, exposure period, geographic area, sample size, quality score, publication year, and adjustment for potential confounders (yes or no). In addition, we performed a sensitivity analysis by removing omitting one study at a time and calculating the overall RR for the remaining studies. A dose-response meta-analysis was conducted to explore the trend between folate intake and asthma risk. Furthermore, we used generalized least-squares trend (GLST) estimation to calculate the trend from the relevant log-RR estimates across folate intake category^23,24^. The publication bias was assessed by Egger’s test and Begg’s visual inspection of funnel plots^25,26^. All statistical tests were two-sided and performed by STATA software (version 11.2, StataCorp, College Station, TX, USA). *P* values < 0.05 were considered significant.

## Results

### Literature search

**Figure 1:**
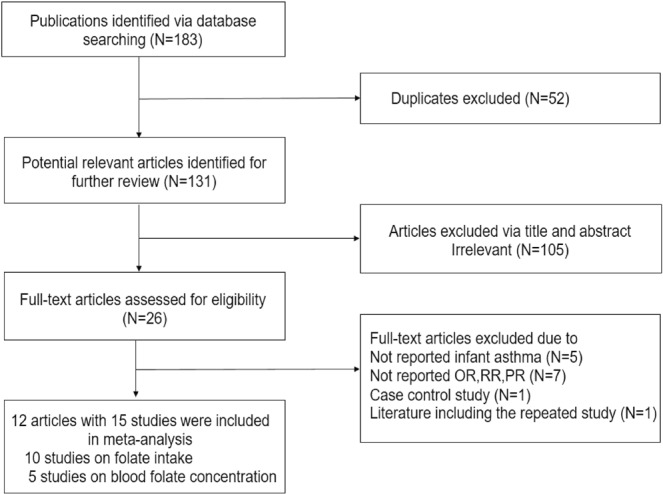
Flowchart of the search strategy and study selection process.

The flow chart with the literature selection details is presented in Fig. 1. In total, 26 articles were considered for further estimation: 5 articles did not report on infant asthma; 7 articles did not report ORs, RRs, or PRs; and 2 articles included a repeated study, and we chose the latest one and excluded a case control study^27^. Finally, 12 articles^28–38^ with 10 studies^28–36,39^ on folate intake and 5 studies^29,30,35,37,38^ on blood folate concentration were included in our meta-analysis. In addition, the data of the Avon longitudinal birth cohort^39^ were derived from a review^40^.

### Study characteristics

The information of the 10 studies assessing folate intake is shown in Table 1. These studies were published from 2008 to 2018 and included 201,248 participants. Seven studies were conducted in Europe^29,30,32,33,35,39,41^, two in North America^31,34^, and one in Australia^36^. Among the 10 studies that reported folate intake, 5 studies were related to supplemental folate intake^29,31–33,35^, and 5 studies were related to total folic acid intake from diet and supplements^28,30,34,36,39^. The studies included in the analysis were adjusted for a number of potential confounders, such as maternal age, maternal smoking, maternal asthma history, infant gender. The characteristics of 5 studies focusing on blood folate concentrations were summarized in Table 2. The details of the NOS evaluation method are shown in Supplementary Table 1. Consequently, four studies received 6 stars, five studies received 7 stars, and one study received 8 stars.

**Table 1:** Baseline characteristics of individual studies on maternal folate intake and infant asthma.

Author, year	Region	Study type	Sample size	Mothers’ age (years)	Sources of folic acid intake	Exposure period	Age at visit (years)	Assessments of outcomes	Adjustment for covariates	Study quality^#^
Trivedi M. K. *et al*.^27^ 2018	United States	Cohort study	1,279	32.2	Foods and supplements	First trimesters, second trimester	7.9	Based on the validated instruments from the ISAAC*	Maternal age, maternal history of asthma, household income, child race/ethnicity, gestational age, breastfeeding duration, and age at mid-childhood visit	7
den Dekker H. T. *et al*.^28^ 2018	Netherlands	Cohort study	5,653	31	Folic acid supplementation use	Preconception, before 18 weeks of gestation	10	Physician ever having diagnosed asthma or the use of inhalant medication in the past 12 months	Maternal age, maternal history of asthma, child race/ethnicity, gestational age, body mass index at intake, parity, educational level, smoking or alcohol use	7
Parr C. L. *et al*.^29^ 2017	Norwegian	Cohort study	39,846	30.0	Foods and supplements	18 and 22 weeks of pregnancy	7	Use at least two asthma medications or maternal report of the child ever having physician-verified asthma plus either asthma symptoms or asthma medication use in the past year	Parity, maternal education, prepregnancy body mass index, maternal history of atopy, maternal smoking in pregnancy, use of cod liver oil or other dietary supplements, and maternal energy intake in pregnancy	7
Veeranki S. P. *et al*.^30^ 2015	United States	Cohort study	104,428	22	Folic acid-containing supplements	First trimester only, after first trimester, first trimester and beyond	4.5–6	Using a previously validated algorithm that uses asthma-specific healthcare visits and asthma-specific medication use	Infant gender, estimated gestational age, birth weight, other living siblings, maternal race, region of residence, pregnancy year, marital status, age at delivery, level of education, smoking during pregnancy and adequacy of prenatal care	7
Zetstra-van der Woude P. A. *et al*.^31^ 2014	Netherlands	Cohort study	35,604	15–50	Folic acid supplements	During pregnancy.	Childhood	Use of asthma medication	Maternal age, dispensation of benzodiazepines during pregnancy, and maternal dispensation of asthma medication	8
Bekkers M. B. *et al*.^32^ 2012	Netherlands	Cohort study	3786	30.5	Folic acid supplements	During pregnancy.	1–8	At least one attack of wheeze, and/or at least one attack of dyspnoea, and/or prescription of inhalation steroids for respiratory or lung problems by a medical doctor	Maternal education, maternal allergy, maternal smoking during pregnancy and number of older siblings	6
Martinussen M. P. *et al*.^33^ 2012	USA	Cohort study	1499	<25: 22.75%; 25–35: 58.97%; >35: 18.28%	Food and supplements	First trimester, 1 month before conception	6	Mothers’ reports of physician-diagnosed asthma or wheezing or whistling symptoms ever in the last 12 months	Household annual income, maternal marital status, and physician diagnosed maternal asthma	6
Magdelijns F. J. *et al*.^34^ 2011	Netherlands	Cohort study	2640	NA	Folic acid supplements	4 weeks before until 8 weeks after conception	6–7	Physician-diagnosed asthma with clinical symptoms and/or the use of asthma medication ever in the last 12 months	Maternal antibiotic use during pregnancy, maternal smoking and alcohol consumption during pregnancy, mode and place of delivery, birth weight, infant gender, treatment with antibiotics during the first 6 months of life, breastfeeding during the first 2 years of life, exposure to domestic animals during pregnancy and the first 2 years of life, exposure to environmental tobacco smoke in the first 6 to 7 years of life, siblings, family history, recruitment group, maternal education level, day care, and other supplement use during pregnancy	7
Whitrow M. J. *et al*.^35^ 2009	Australia	Cohort study	423	30.5	Food and supplements	Prepregnancy; <16 weeks; 30–34 weeks	5.5	Physician-diagnosed asthma or current asthma	Maternal education, maternal age, parity, gestational age, maternal asthma status, and breastfeeding	6
Granell R. *et al*.^38^ 2008	UK	Cohort study	6090	28.4	Food and supplements	18 and 32 weeks of pregnancy.	7.5	Physician diagnosed asthma and wheezing during the past 12 months	Gender, maternal history of asthma or allergy, maternal dietary folate intake at 18 or 32 weeks gestation, exposure to prenatal and postnatal maternal smoking and maternal education	6

*ISAAC: the International Study of Asthma and Allergies in Childhood.

^#^Quality assessment was performed with the NOS.

**Table 2:** Baseline characteristics of individual studies on blood folate concentration and infant asthma.

Author	Year	Region	N	RR (95%CI)	Sample type	Adjustment for covariates
den Dekker H. T. *et al*.^29^	2018	Netherlands	276	0.93 (0.79–1.09)	Serum	Maternal age and BMI at intake, parity, history of asthma or atopy, educational level, smoking or alcohol use during pregnancy, child’s gestational age at birth, birthweight and ethnicity
Parr C. L. *et al*.^30^	2017	Norway	2,681	0.97 (0.54–1.76)	Plasma	Maternal age at delivery, parity maternal education, prepregnancy BMI, maternal history of atopy, maternal smoking in pregnancy, use of cod liver oil and other dietary supplements in pregnancy, and gestational week of sample collection
Magdelijns F. J. *et al*.^35^	2011	Netherlands	2640	0.31 (0.09–1.10)	In erythrocytes	Maternal antibiotic use during pregnancy, maternal smoking during pregnancy, maternal alcohol consumption during pregnancy, mode and place of delivery, birth weight, gender of the child, treatment with antibiotics during the first 6 months of life, breastfeeding duration, exposure to domestic animals during pregnancy and the first 2 years of life, exposure to environmental tobacco smoke in the first 6 to 7 years of life, siblings, family history of atopy, recruitment group, maternal education level, day care, and multivitamin or other supplement use during pregnancy.
van der Valk R. J. *et al*.^37^	2013	Netherlands	2,001	1.02 (0.83–1.25)	Cord blood	Maternal age, BMI, educational level at intake, history of maternal atopy or asthma, smoking and folic acid supplement use during pregnancy, parity and children’s sex, gestational age and birth weight
Håberg S. E. *et al*.^38^	2011	Norway	1962	1.66 (1.16–2.37)	Plasma	Maternal educational level, maternal age, parity, maternal atopy, maternal BMI, maternal smoking in pregnancy and maternal smoking at age 3 years, supplement use at age 3 years

### Maternal folate intake, blood folate concentration, and infant asthma risk

**Figure 2:**
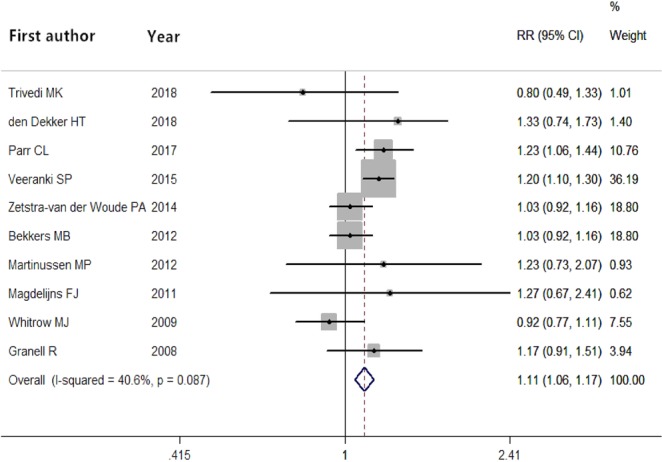
Forest plot showing pooled relative risks and corresponding 95% CIs of infant asthma according to maternal folate intake. The grey squares indicate study-specific relative risks, the horizontal lines represent the 95% CI, and the size of each square is proportional to its weight in the analysis. The diamond represents the summary relative risk estimate with its 95% CI.

The adjusted RRs of maternal folic acid intake and the risk of infant asthma for each study are shown in Fig. 2. The relationship between maternal folate intake during pregnancy and childhood asthma risk is inconsistent. In short, a summary RR of maternal folate intake was 1.11 in the fixed effects model (95% CI = 1.06–1.07; *P* = 4.664 × 10^−5^), revealing that maternal folate intake during pregnancy was significantly associated with the risk of infant asthma. We did not conduct meta-regression analyses to identify the sources of heterogeneity due to the low heterogeneity (*P* = 0.087).

**Figure 3:**
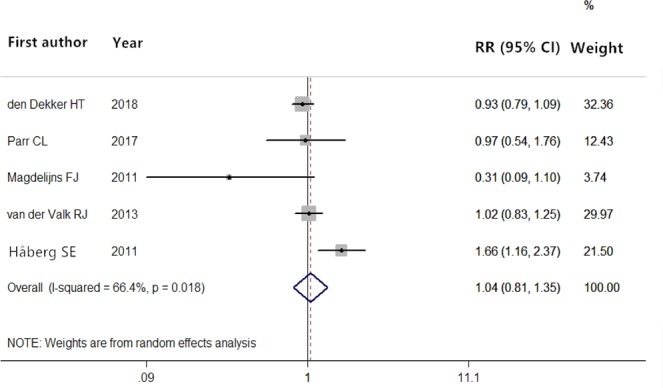
Forest plot showing pooled relative risks and corresponding 95% CIs of infant asthma according to blood folate concentration.

In addition, 5 studies^29,30,35,37,38^ reported the blood folate concentration. Figure 3 shows the RRs for the association of maternal blood folate concentration with infant asthma risk. The heterogeneity of the results was high (*P* = 0.018), and the pooled RR was 1.04 (95% CI = 0.81–1.35; *P* = 0.737) in the random models compared with the reference category.

In the subgroup analyses of geographic region, we found a significantly increased risk of folic acid intake for Europe (RR = 1.08; 95% CI = 1.01–1.16) and North America (RR = 1.20; 95% CI = 1.11–1.30) (Table 3). The analysis by publication year yielded pooled RRs of 1.03 (95% CI = 0.94–1.12) in 5 studies published before 2013 and 1.15 (95% CI = 1.08–1.22) in 5 studies published after 2013. In addition, the stratified analysis by sample size revealed pooled RRs of 1.00 (95% CI = 0.91–1.10) in 5 studies with under 5000 participants and 1.16 (95% CI = 1.09–1.23) in 5 studies with more than 5000 participants. Furthermore, when we stratified the analysis by quality score, the RR was 1.02 (95% CI = 0.94–1.12) in 4 studies with scores <7, while the RR was 1.15 (95% CI = 1.08–1.22) in 6 studies with scores ≥7. In the stratification studies of folate source intake and asthma risk, we found that folate intake from supplements increased the infant asthma risk (RR 1.12; 95% CI = 1.05–1.18), while the effect was not significant for folate intake from diet and supplements (RR = 1.09; 95% CI = 0.99–1.21).

**Table 3:** Results of the subgroup analysis for the association between maternal folate intake and infant asthma risk.

Variables	N	RR (95%CI)^a^			Heterogeneity test^b^		
		Estimate	95% CI		χ^2^	*P*	I^2c^
All studies		1.11	1.06	1.17	15.16	0.087	40.60%
Geographic region							
Europe	7	**1**.**08**	**1**.**01**	**1**.**16**	7.00	0.321	14.23%
North America	2	**1**.**20**	**1**.**11**	**1**.**30**	0.01	0.927	0.00%
Australia	1	0.92	0.77	1.11	—	—	—
Publication year							
<2013	5	1.03	0.94	1.12	3.29	0.510	0.00%
≥2013	5	**1**.**15**	**1**.**08**	**1**.**22**	7.69	0.104	48.00%
Sample size							
<5000	5	1.00	0.91	1.10	2.96	0.565	0.00%
≥5000	5	**1**.**16**	**1**.**09**	**1**.**23**	5.63	0.228	29.00%
Quality score							
<7	4	1.02	0.94	1.12	2.87	0.413	0.00%
≥7	6	**1**.**15**	**1**.**08**	**1**.**22**	7.78	0.169	35.70%
Folate source							
Total^d^	5	1.09	0.99	1.21	7.67	0.104	47.80%
Supplement	5	**1**.**12**	**1**.**05**	**1**.**18**	7.39	0.117	45.90%
Exposure period							
Early pregnancy	7	**1**.**15**	**1**.**07**	**1**.**23**	9.37	0.154	36.0%
Others	3	1.08	0.97	1.20	4.01	0.135	50.1%
Assessment method							
FFQ	5	1.09	0.99	1.21	7.67	0.104	47.80%
Others	5	**1**.**12**	**1**.**05**	**1**.**18**	7.39	0.117	45.90%
Adjustments maternal smoking							
Yes	6	**1**.**16**	**1**.**09**	**1**.**23**	5.71	0.335	12.50%
No	4	1.00	0.91	1.10	2.42	0.491	0.00%
Adjustments maternal allergy							
Yes	8	1.06	1.00	1.13	9.61	0.212	27.20%
No	2	**1**.**20**	**1**.**11**	**1**.**31**	0.03	0.863	0.00%
Adjustments maternal education							
Yes	7	**1**.**13**	**1**.**07**	**1**.**20**	11.23	0.081	46.60%
No	3	1.03	0.92	1.15	1.42	0.491	0.00%

RR: relative risk FFQ: Food Frequency Questionaire.

^a^RR (95% Cl) indicates pooled estimates of study-specific RRs with corresponding 95% CIs.

^b^Heterogeneity test indicates the heterogeneity of subgroup analyses.

^c^I^2^ shows the degree of heterogeneity among studies.

^d^Total folic acid intake was from diet and supplements

### Sensitivity analyses

Sensitivity analyses indicated that the overall RR was not markedly influenced by the removal of any single study except for Veeranki S. P. *et al*.^31^. (Supplementary Fig. 1).

### Dose-response relationship between folate intake and asthma

**Figure 4:**
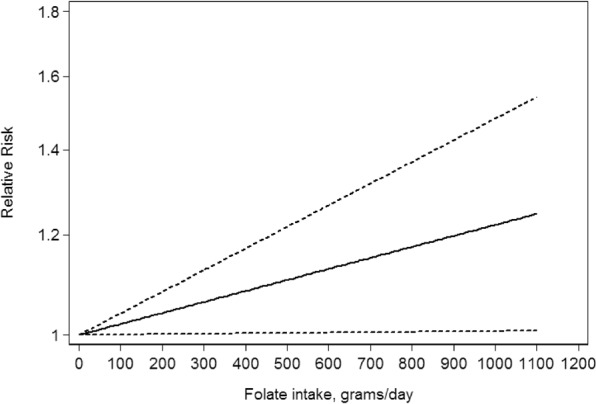
Dose-response relationship between maternal folate intake and infant asthma. The solid line and the dash line represent the estimated relative risks and corresponding 95% confidence intervals. Folic acid intake was modeled with a linear trend (P-value for non-linearity = 0.82) in a fixed-effects model.

Studies with available related data were selected to conduct a dose-response analysis^30,34^. The dose-response relationship between maternal folate intake and infant asthma is shown in Fig. 4. Because no evidence of departure from linearity was found (*P* = 0.824), we finally assumed a linear relationship in a fixed-effect model (*P*_heterogeneity_ = 0.055) with a linear dose-response relationship (*P*_linearity_ = 0.042). The result of our dose-response analysis suggests that each 100-mg/day increment in maternal folate intake was associated with a 0.02% higher risk of infant asthma.

### Publication bias

No publication bias was noted with Egger’s test (*P* = 0.788) or Begg’s funnel plot (Supplementary Fig. 2).

## Discussion

The results of this meta-analysis suggested that maternal folate intake is associated with an increased risk of infant asthma. In stratified analyses, we found that such an effect was significant in Europe and North America. The influence remains statistically significant in studies with later years of publication or larger sample sizes or in studies of higher quality.

The study of the relationship between folate intake during pregnancy and asthma during pregnancy was first performed by Granell R. *et al*. in^39^. After that, several studies were conducted to further explore the relationship, but their results are not consistent. Thus, Krista S. Crider *et al*.^22^ conducted a meta-analysis including 5 studies and ultimately provided no evidence of a significant relationship between maternal folic acid supplement use and infant asthma in offspring (RR = 1.01; 95% CI = 0.78–1.30). Liu Yang *et al*.^27^ also performed a meta-analysis including 5 studies in 2015 and found that the connection between maternal folate intake during pregnancy and infant asthma risk was not significant (RR = 1.06, 95% CI = 0.99–1.14). Afterwards, Parr C. L. *et al*.^30^ and Veeranki S. P. *et al*.^31^ conducted corresponding cohort studies, and found a significant relationship between folic acid intake during pregnancy and infant asthma. Given the increasing number of studies on the relationship between maternal folate intake and infant asthma, we included more literature for further meta-analysis to more thoroughly explore this relationship and to find a significant association between maternal folate intake and infant asthma. In addition, several differences between our meta-analysis and the previous analyses were markedly observed. First, the previous analyses included no more than 5 studies, and the publication year was limited to 2012. Our analysis included 10 studies related to maternal folate intake and 5 studies related to blood folate concentration. Our meta-analysis is more statistically convincing than previous studies due to a relatively larger number of studies and sample sizes. Second, a previous meta-analysis on prenatal folate intake and infant asthma only summed the available evidence qualitatively. In our analysis, we made full use of the available dose data and performed a dose-response analysis, which quantitatively reveals the relationship between maternal folic acid intake during pregnancy and the risk of infant asthma. Last, we further conducted subgroup analyses based on the characteristics of included studies, such as analyses by geographic region, publication year, folate source and other significant factors.

The mechanism by which folic acid affects the development of asthma in children may be achieved via variable DNA methylation in the mother’s uterus. In DNA methylation, a methyl group is transferred from s-adenosylmethionine to cytosine by the action of a transmethylase, which plays a vital role in regulating cell growth^42^. Hollingsworth *et al*. found that Runt-related transcription factor 3 (Runx3), a gene known for the prevention of allergic airway disease, was excessively methylated in progeny exposed to a high-methylation diet, resulting in the suppression of Runx3 mRNA and protein levels. Methyl sources are supplemented in pregnant mice to alter DNA methylation and to ultimately predisposed the mice to allergic airway disease by inducing the differentiation of T-lymphocytes to a TH2 phenotype^43^. Subsequently, a serious number of epidemiologic studies were performed to assess the potential link between maternal folate intake and infant asthma. However, we recognized that the underlying mechanism of folate-induced infant asthma is still limited.

Several strengths were observed in our study. First, our analysis was based on a comprehensive bibliographic search including 201,248 participants, which provide sufficient statistical power for our research. Second, subgroup analyses of included studies were conducted by geographic region, publication year, folate source, and other factors, which indicated the influence of different covariates on maternal folate intake and infant asthma risk. Finally, there was no publication bias in our analysis. However, the limitations of this analysis should be considered in the interpretation of our findings. First, all the studies included in our analysis were adjusted for known infant asthma risk factors, but these factors were not consistent. Second, it is difficult to accurately determine how much folate in natural food and in its synthetic form was consumed (used in multivitamins, prenatal fortified supplement) during pregnancy. Because of misclassifications of folate sources or inaccurate measurements of blood folate concentration, the included studies may have potential bias. Third, the dose-response analysis demonstrated a linear association between maternal folate intake and asthma risk; however, due to the lack of included studies, more dose-response studies are needed to further confirm this linear relationship.

Our meta-analysis showed that maternal folate intake during pregnancy increases the risk of infant asthma. Meanwhile, the dose-response analysis confirmed a linear association between maternal folic acid intake and the risk of infant asthma. Therefore, the adverse effect of folic acid on infant asthma should not be ignored when it was supplemented during pregnancy to prevent birth defects. Further studies are warranted to determine a critical intake dose of folate in pregnancy that can effectively prevent the adverse effect of infant asthma while also preventing birth defects.

## Supplementary information

Supplementary Tables and Figures

## Data Availability

All data included in this study are available upon request by contact with the corresponding author.
