# Supplementary material for: Association of maternal folate intake during pregnancy with infant asthma risk
Source: Sci Rep. 2019 Jun 6;9:8347. doi: 10.1038/s41598-019-44794-z (PMC6554315; doi:10.1038/s41598-019-44794-z)
Supplement: Supplementary file 1 — Supplementary Tables and Figures [file 41598_2019_44794_MOESM1_ESM.pdf]

Association of maternal folate intake during pregnancy with infant asthma risk

Weijian Li[1, 2†], Bo Xu[3†], Yuepeng Cao[2], Yang Shao[4], Wanke Wu[1], Jun Zhou[2], Xiaofang Tan[1], Xiaoli Wu[1], Jing Kong[1], Chen Hu[1], Kaipeng Xie [1*], Jiangping Wu[1*]

[1]The Affiliated Obstetrics and Gynecology Hospital of Nanjing Medical University, Nanjing Maternity and Child Health Care Hospital, Women's Hospital of Nanjing Medical University, Nanjing 210004, China

[2]Department of Urology, Drum Tower Hospital of Nanjing University Medical School, Nanjing 210008, China

[3]State Key Laboratory of Reproductive Medicine, Institute of Toxicology, Nanjing Medical University, 211166, China

[4]The First People's Hospital of Zhangjiagang city, The Zhangjiagang Affiliated Hospital of Soochow University, Suzhou 215600, China

[†] These authors contributed equally to this work.

[*]**Corresponding author**: Jiangping Wu, The Affiliated Obstetrics and Gynecology Hospital of Nanjing Medical University, Nanjing Maternity and Child Health Care Hospital, Women's Hospital of Nanjing Medical University, Nanjing, 210004, China, Tel: +86-25-5222-6472, Fax: +86-25-5222-6472, E-mail: wujiangping@njmu.edu.cn Or Kaipeng Xie, Nanjing Maternity and Child Health Care Institute, The Affiliated Obstetrics and Gynecology Hospital of Nanjing Medical University, Nanjing Maternity and Child Health Care Hospital, Nanjing 210004, China. Tel: +86-25-5222-6264, Fax: +86-25-522-26264, E-mail: kaipengxie@njmu.edu.cn.

Supplementary table 1.Quality of articles included in the analysis

Article (authors)	Year	Selection				Comparability		Outcome			NOS
Conditions		1	2	3	4	a	b	1	2	3	score
Trivedi MK *et al* [27]	2018	*	*	-	*	-	*	*	*	*	7
den Dekker HT *et al* [28]	2018	*	*	-	*	*	-	*	*	*	7
Parr CL *et al* [29]	2017	*	*	-	*	*	-	*	*	*	7
Veeranki SP *et al* [30]	2015	*	*	*	-	*	-	*	*	*	7
Zetstra-van der Woude PA *et al* [31]	2014	*	*	*	*	*	-	*	*	*	8
Bekkers MB *et al* [32]	2012	*	*	-	*	-	*	-	*	*	6
Martinussen MP *et al* [33]	2012	*	*	-	*	*	-	-	*	*	6
Magdelijns FJ *et al* [34]	2011	*	*	-	*	*	-	*	*	*	7
Whitrow MJ *et al* [35]	2009	*	*	-	*	-	*	-	*	*	6
Granell R *et al* [38]	2008	*	*	-	*	-	*	-	*	*	6

Note: ‘*’ indicates NOS quality assessment star awarded， ‘-’ indicates that no star was awarded.

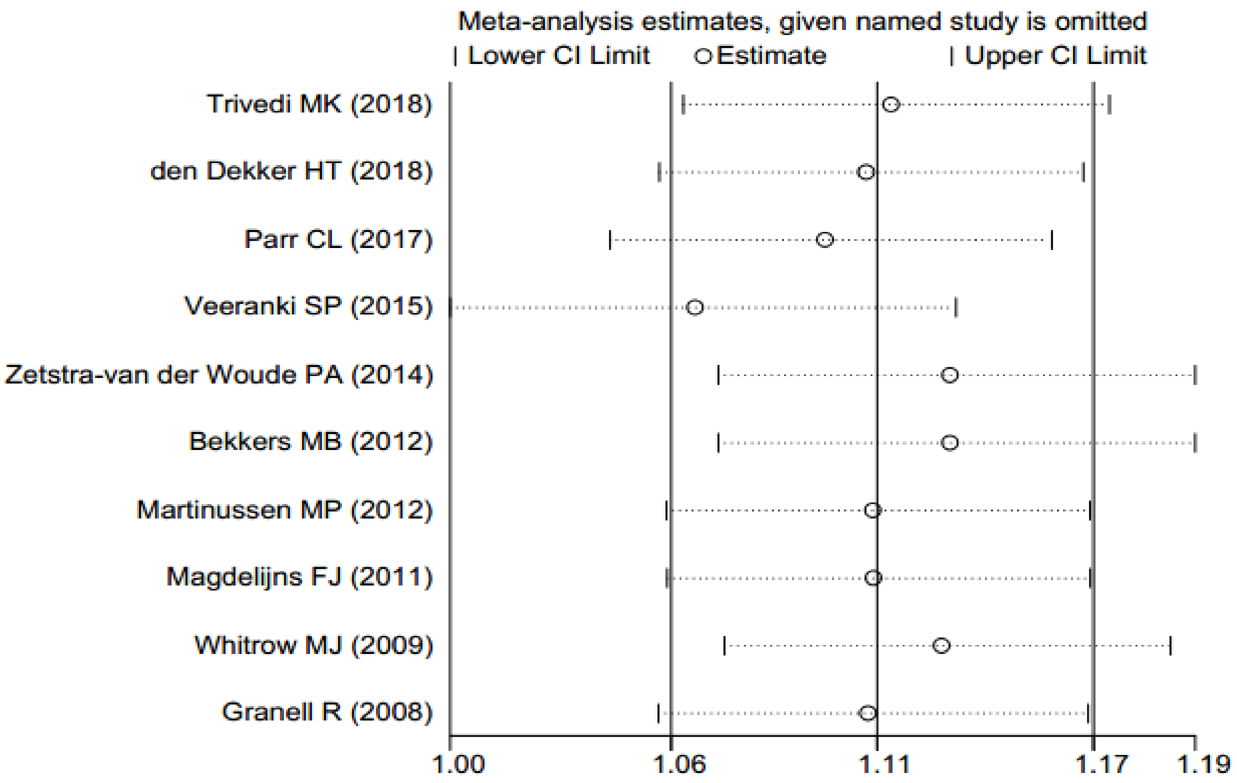

Supplementary Figure 1. Sensitivity analyses indicated that the overall RR was not markedly influenced by removing any single study except for Veeranki, S.P. *et al.*

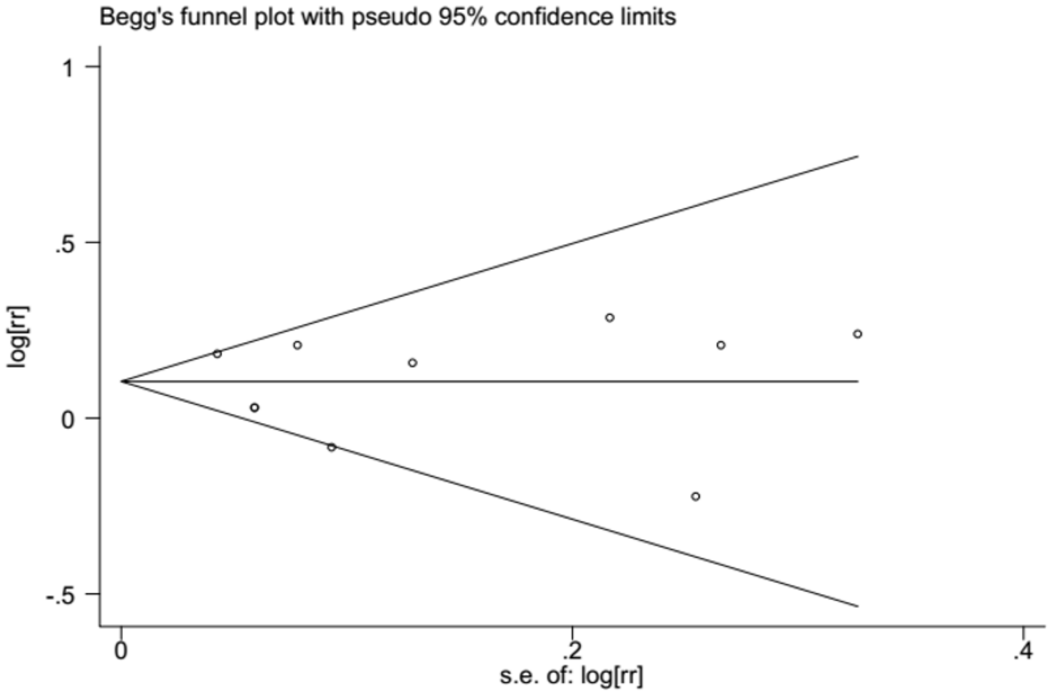

Supplementary Figure 2. Begg's Funnel plot for publication bias of maternal folate intake and infant asthma.
